# Routine cine cardiovascular magnetic resonance for clinical phenotyping and serial assessment of heart failure with preserved ejection fraction: a retrospective single-center clinical analysis

**DOI:** 10.3389/fcvm.2026.1965415

**Published:** 2026-10-19

**Authors:** Jing Wang, Yingjie Xie, Chaoqiang Zeng, Lin Nie, Yang Yang

**Affiliations:** 1 Department of Imaging, Beijing Anzhen Nanchong Hospital, Capital Medical University and Nanchong Central Hospital, Nanchong, China; 2 One Department of Cadre Ward, General Hospital of Southern Theater Command of Chinese PLA, Guangzhou, China; 3 Department of Radiology, Ya'an People’s Hospital, Ya'an, Sichuan, China

**Keywords:** cardiovascular magnetic resonance, cine imaging, global longitudinal strain, heart failure with preserved ejection fraction, left atrial volume index, penalized logistic regression, serial imaging

## Abstract

**Background:**

Heart failure with preserved ejection fraction (HFpEF) is difficult to confirm because symptoms are non-specific, comorbidities are prevalent, and left ventricular ejection fraction (LVEF) is normal by definition. We evaluated whether markers from routine cine cardiovascular magnetic resonance (CMR) improved phenotyping and diagnostic discrimination in HFpEF and whether they changed after treatment optimization.

**Methods:**

In this retrospective single-center, observational analysis, 126 adults evaluated between January 2023 and December 2025 were included: 72 with adjudicated HFpEF, 27 hypertensive controls without heart failure, and 27 healthy volunteers. All had available 1.5-T cine CMR studies that underwent blinded analysis of LV volumes, LVEF, left ventricular mass index (LVMI), left atrial volume index (LAVI), right ventricular ejection fraction, and feature-tracking global longitudinal strain (GLS). Interobserver reproducibility was quantified. The primary analysis estimated the discrimination of LAVI, LVMI, and GLS for HFpEF; a multiparametric model was developed using Firth-penalized logistic regression with bootstrap optimism correction. Forty-six patients with HFpEF had repeat CMR available at approximately 6 months after individualized treatment optimization and were included in the serial analysis.

**Results:**

LVEF was preserved and similar across groups. HFpEF was associated with higher LVMI, marked LAVI enlargement, and less negative GLS, with large effect sizes for LAVI and GLS. LAVI and GLS were the strongest single discriminators [apparent area under the curve (AUC) 0.83 and 0.85, respectively]; the multiparametric model achieved an apparent AUC of 0.87 that fell to 0.84 after optimism correction and did not exceed GLS alone. LAVI and less negative GLS remained independent predictors after adjustment. At 6 months, LAVI decreased (mean change −4.8 mL/m^2^), and GLS became more negative (−1.3 percentage points), exceeding measurement variability, whereas LVEF was unchanged.

**Conclusions:**

Routine cine CMR identified atrial remodeling, ventricular hypertrophy, and impaired longitudinal deformation despite preserved LVEF, and appeared capable of detecting selective short-term temporal changes rather than definitive treatment-induced reverse remodeling. It is a practical adjunct for phenotyping and hypothesis-generating serial assessment, pending external validation.

## Introduction

Heart failure with preserved ejection fraction (HFpEF) accounts for a large and growing proportion of heart failure cases and remains among the most challenging syndromes in cardiovascular practice (1, 2). The diagnostic difficulty is fundamentally clinical: patients present with dyspnea, fatigue, exercise intolerance, or congestion, yet left ventricular ejection fraction (LVEF) is normal or near-normal by definition (3). Contemporary consensus holds that heart failure requires symptoms or signs arising from a structural or functional cardiac abnormality, supported by elevated natriuretic peptide concentrations or objective evidence of congestion (4). In HFpEF, this construct is complicated by obesity, hypertension, diabetes, atrial fibrillation, pulmonary and renal disease, deconditioning, and aging, all of which can mimic or amplify heart failure physiology (5–7).

Echocardiography is the first-line imaging modality for suspected HFpEF because it is accessible, dynamic, and embedded in validated diagnostic frameworks such as HFA-PEFF and H_2_FPEF (8–10). Nevertheless, diastolic indices may be discordant, image quality is frequently limited in patients with obesity or lung disease, and LVEF does not capture clinically important abnormalities in myocardial mechanics, atrial remodeling, or filling-pressure burden. These limitations are most consequential in the population in which HFpEF predominates, namely older patients with multiple cardiometabolic comorbidities and overlapping causes of dyspnea (5, 6). A clinically useful imaging strategy should therefore clarify the phenotype, distinguish cardiac from non-cardiac contributors where possible, and provide markers that are reproducible enough for serial assessment.

Cardiovascular magnetic resonance (CMR) quantifies ventricular volumes, myocardial mass, and global function with high reproducibility and without geometric assumptions (11, 12). Within the broader spectrum of contemporary CMR applications in heart failure, which encompasses ventricular volumetric and mass quantification, feature-tracking strain analysis, tissue characterization by late gadolinium enhancement and parametric mapping, stress perfusion, and etiological assessment (13), cine imaging represents the foundational sequence on which more elaborate protocols are built. Balanced steady-state free-precession cine imaging is particularly attractive for pragmatic HFpEF assessment because it is already part of routine protocols, requires no contrast agent, and can be post-processed to estimate left atrial volume and myocardial deformation (11, 12). Left atrial CMR markers, including left atrial volume index (LAVI) and atrial strain, identify HFpEF at least as accurately as many ventricular indices (14, 15), including in hypertensive populations in whom the distinction is most difficult (16), and atrial roof remodeling has emerged as a distinctive structural signature (17). Feature-tracking global longitudinal strain (GLS) is associated with invasive diastolic dysfunction and with adverse outcomes despite preserved LVEF (18–20), and longitudinal decline in left atrial function precedes incident HFpEF in community cohorts (21). These observations are clinically coherent: left atrial enlargement integrates chronic filling-pressure exposure, whereas GLS reflects subclinical longitudinal dysfunction that ejection fraction does not reveal.

Advanced tissue characterization, including T1 mapping, extracellular volume quantification, stress perfusion, and late gadolinium enhancement, has refined mechanistic understanding and may aid risk stratification (22–24). However, these techniques are not universally available, add protocol complexity, and may be impractical or contraindicated in some settings. A cine-first analysis therefore addresses a pragmatic question: whether routinely available cine-derived markers can define a reproducible HFpEF phenotype and provide objective serial markers after treatment optimization. We designed this retrospective, single-center study to compare cine CMR findings among adjudicated HFpEF patients, hypertensive controls, and healthy volunteers, and to evaluate whether LAVI, left ventricular mass index (LVMI), and GLS provide information incremental to preserved ejection fraction.

## Materials and methods

### Study design, ethics, and reporting

This retrospective single-center, observational clinical analysis was conducted at a tertiary teaching hospital. The protocol was approved by the Institutional Ethics Committee of Nanchong Central Hospital, Southern Branch of Beijing Anzhen Hospital, Capital Medical University [Approval No. 2026(160)] before data collection and analysis, and was performed in accordance with the Declaration of Helsinki (25). Participant data were de-identified before analysis. The report follows the Strengthening the Reporting of Observational Studies in Epidemiology (STROBE) recommendations, and the diagnostic model component is reported in accordance with the Transparent Reporting of a multivariable prediction model for Individual Prognosis Or Diagnosis (TRIPOD + AI) guidance (26, 27). The requirement for individual informed consent was waived by the Institutional Ethics Committee owing to the retrospective study design.

### Participants and cohort definitions

Consecutive adults evaluated for exertional dyspnea or suspected HFpEF between January 2023 and December 2025 were identified from institutional clinical and imaging records. Hypertensive controls and healthy volunteers with available cine CMR examinations performed during the same period were identified from institutional records as clinically relevant comparator groups. HFpEF status was adjudicated by two cardiologists using prespecified criteria based on contemporaneously recorded clinical, biomarker, and echocardiographic data, and disagreements were resolved by consensus. CMR readers were blinded to cohort assignment and to clinical data other than the age and sex required for identity verification. The participant flow and the analytic structure are summarized in Figure 1.

**Figure 1:**
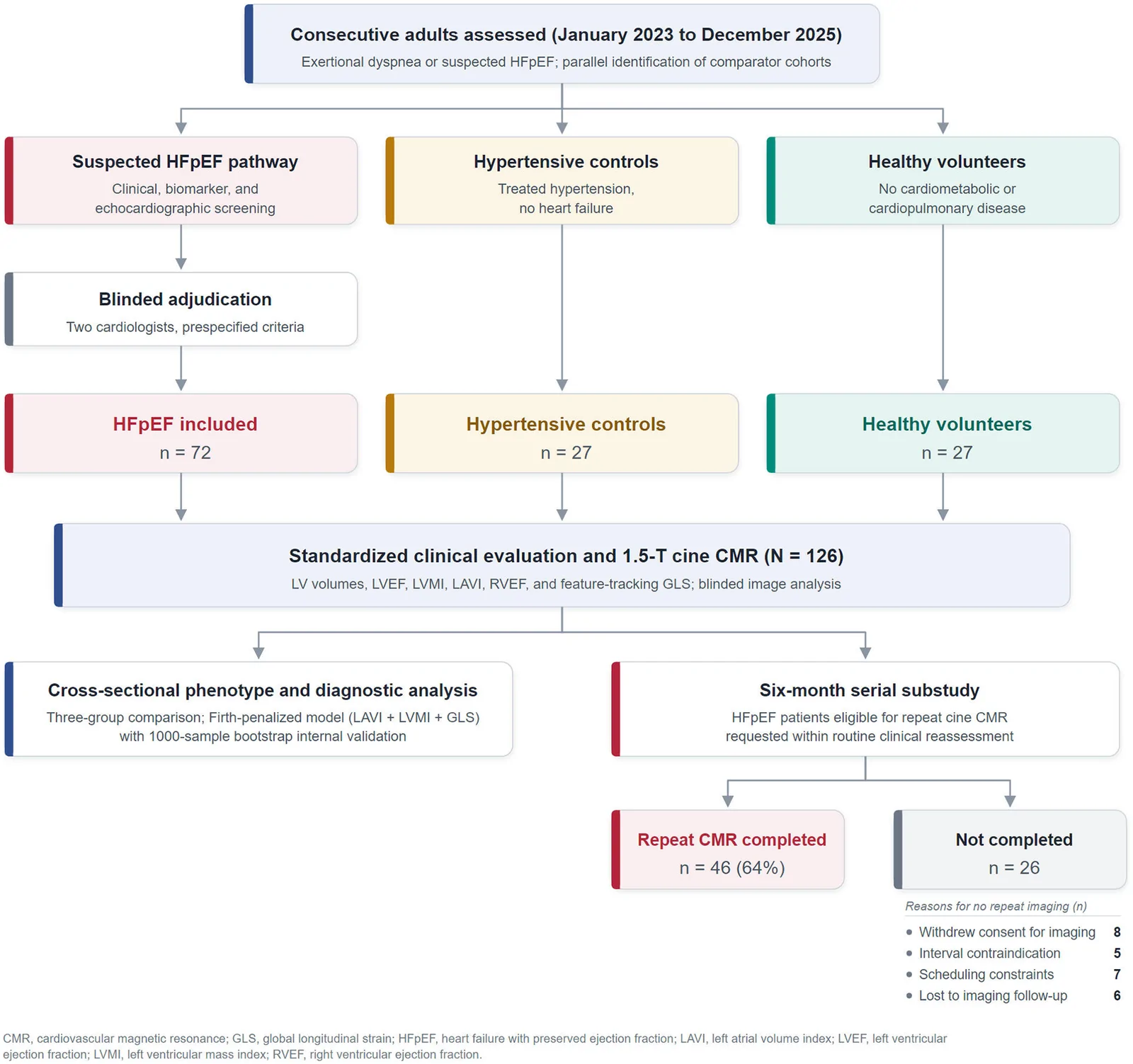
Retrospective cohort identification and analytic workflow. Consecutive adults assessed for exertional dyspnea or suspected HFpEF and parallel comparator cohorts are shown, with blinded adjudication, the included groups (HFpEF, *n* = 72; hypertensive controls, *n* = 27; healthy volunteers, *n* = 27), standardized 1.5-T cine CMR in all 126 participants, cross-sectional phenotype and diagnostic analyses, and the 6-month serial substudy with completion, attrition, and reasons for the absence of repeat imaging.

### Eligibility criteria

Eligibility criteria were applied retrospectively to existing clinical and imaging records for each cohort. All diagnoses were confirmed from source documentation before CMR analysis, and natriuretic peptide concentrations were interpreted according to cardiac rhythm. The inclusion and exclusion criteria are detailed below.

#### Inclusion criteria for the HFpEF cohort

- Age of at least 18 years with symptoms or signs of heart failure, including exertional dyspnea, fatigue, reduced exercise tolerance, or congestion.
- Left ventricular ejection fraction of at least 50% on transthoracic echocardiography.
- Elevated natriuretic peptides using rhythm-specific thresholds (NT-proBNP greater than 125 pg/mL in sinus rhythm or greater than 365 pg/mL in atrial fibrillation).
- Objective evidence of a structural or functional cardiac abnormality, such as left atrial enlargement, left ventricular hypertrophy, or echocardiographic indices of elevated filling pressure consistent with an intermediate-to-high HFA-PEFF profile (4, 8–10).

#### Inclusion criteria for hypertensive controls

- Documented or pharmacologically treated systemic hypertension.
- Preserved left ventricular ejection fraction with no prior or current diagnosis of heart failure.
- Absence of clinical congestion at the index assessment.

#### Inclusion criteria for healthy volunteers

- Absence of known cardiovascular disease, hypertension, diabetes mellitus, chronic pulmonary disease, chronic kidney disease, and heart failure symptoms.

#### Exclusion criteria common to all cohorts

- Left ventricular ejection fraction below 50%, or moderate or severe valvular heart disease.
- Infiltrative, hypertrophic, congenital, or constrictive cardiomyopathy.
- Recent acute coronary syndrome, myocarditis, pulmonary embolism, coronary revascularization, or decompensated heart failure.
- Uncontrolled tachyarrhythmia precluding adequate image quality.
- Any contraindication to CMR examination, or, in the healthy-volunteer cohort, inability to provide informed consent for the study CMR.

### Clinical and echocardiographic assessment

Baseline demographic, clinical, laboratory, and medication data were extracted retrospectively from the electronic medical record using a standardized case report form capturing age, sex, body mass index, blood pressure, resting heart rate, cardiovascular risk factors, diabetes mellitus, atrial fibrillation, New York Heart Association (NYHA) functional class, medications, serum creatinine, and NT-proBNP. Transthoracic echocardiographic data obtained as part of routine care before the index CMR examination were recorded to support diagnostic adjudication and clinical correlation rather than to duplicate the cine CMR analysis.

### CMR acquisition

All included examinations were clinically acquired on a 1.5-T Siemens MAGNETOM Aera (Siemens Healthineers, Erlangen, Germany) system equipped with a dedicated phased-array cardiac receiver coil and retrospective electrocardiographic gating. Following localizer and reference acquisitions, balanced steady-state free-precession cine images were obtained in contiguous short-axis slices spanning the entire left ventricle from the mitral valve plane through the apex, together with standard two-, three-, and four-chamber long-axis views. Representative acquisition parameters were an 8-mm slice thickness, a 2-mm interslice gap, a temporal resolution of 30–45 ms reconstructed to 25–30 cardiac phases, a flip angle of approximately 60°, and an in-plane spatial resolution of approximately 1.5 mm × 1.8 mm, with the field of view adjusted to body habitus. Images were acquired during end-expiratory breath-holds when feasible, and a free-breathing averaged acquisition was used when participants were unable to sustain adequate breath-holds. No gadolinium-based contrast agent was administered for the primary analysis, consistent with the contrast-free, cine-first design, and acquisition followed contemporary standardized CMR protocol recommendations (11, 12).

### CMR image analysis and reproducibility

All images were analyzed offline with cvi42 version 5.14 (Circle Cardiovascular Imaging, Calgary, Canada) by two readers blinded to cohort assignment and to clinical data. Endocardial and epicardial borders were traced at end-diastole and end-systole on the short-axis cine stack according to standardized postprocessing recommendations (11), with consistent inclusion of papillary muscles and trabeculations within the blood pool and their exclusion from myocardial mass. Left ventricular end-diastolic and end-systolic volumes, stroke volume, ejection fraction, and myocardial mass were computed by summation of disks and indexed to body surface area calculated using the Du Bois formula, and right ventricular ejection fraction was derived from manual short-axis contouring. Maximal left atrial volume was measured at ventricular end-systole using the biplane area-length method from the two- and four-chamber views, excluding the pulmonary veins and left atrial appendage, and indexed to body surface area to yield LAVI. Left ventricular GLS was quantified by feature tracking of the long-axis cine, averaging the two-, three-, and four-chamber segmental longitudinal strain, and reported as a negative percentage so that values closer to zero denote worse deformation. Interobserver reproducibility was assessed by independent reanalysis of 25 randomly selected studies and was quantified using two-way random-effects intraclass correlation coefficients, within-subject coefficients of variation, and Bland–Altman analysis. Agreement was high (intraclass correlation coefficients: LAVI 0.95, 95% confidence interval 0.89–0.98; GLS 0.91, 95% confidence interval 0.81–0.96; LVMI 0.93; left ventricular volumes greater than 0.95), within-subject coefficients of variation were 4.8% for LAVI and 6.2% for GLS, and Bland–Altman analysis showed negligible systematic bias (0.4 mL/m^2^ for LAVI and 0.1 percentage points for GLS) with narrow limits of agreement. The minimal detectable change at the 95% level (MDC_95_), calculated as 1.96 times the square root of two times the within-subject standard deviation, was 2.8 mL/m^2^ for LAVI and 1.2 percentage points for GLS, and served as the prespecified threshold for interpreting serial change.

### Treatment optimization and follow-up

After the index baseline imaging examination, patients with HFpEF received individualized clinical management by the treating cardiologist in accordance with contemporary guidance (28–31). Management could include diuretic adjustment for congestion, blood pressure optimization, initiation of a sodium-glucose cotransporter 2 inhibitor when clinically appropriate, rhythm or rate control for atrial fibrillation, and weight- and comorbidity-directed care, supported by trial evidence for sodium-glucose cotransporter 2 inhibition and mineralocorticoid receptor antagonism (32–35) and by imaging evidence of reverse remodeling with these agents (36). Treatment was neither randomized, protocol-mandated, nor assigned by the imaging team. For the serial substudy, patients with a repeat cine CMR examination available at approximately 6 months as part of clinical follow-up were included. The decision to obtain the repeat examination was made by the treating cardiologist as part of routine clinical reassessment rather than mandated by a prespecified imaging protocol, and members of the imaging team took no part in the decision to reimage. The serial analysis was interpreted as exploratory treatment monitoring rather than as evidence of the efficacy of any single therapy.

### Outcomes

The primary analysis estimated the discrimination of cine-derived LAVI, LVMI, GLS, and a multiparametric model for identifying HFpEF relative to the combined non-HFpEF comparator group, with a prespecified sensitivity analysis restricted to hypertensive controls as the more clinically demanding comparator. Secondary analyses examined between-group differences in cine CMR parameters, independent imaging predictors of HFpEF, and paired 6-month changes among patients who completed repeat imaging.

### Sample size considerations

The study cohort reflected all eligible patients identified during the study window rather than a prespecified sample size target. For the leading marker, 72 cases and 54 comparators provide greater than 90% power to reject an area under the receiver operating characteristic curve (AUC) of 0.5 against an expected AUC of 0.80 at a two-sided alpha of 0.05, and yield an expected 95% confidence interval half-width of approximately 0.07 for an AUC of 0.85. The development cohort therefore supports the estimation of discrimination with moderate precision while remaining insufficient for definitive validation.

### Statistical analysis

Continuous variables were summarized as mean with standard deviation or median with interquartile range according to the Shapiro–Wilk test and inspection of distribution plots, and categorical variables as counts with percentages. Three-group comparisons used one-way analysis of variance with Tukey’s honestly significant difference *post hoc* testing for normally distributed variables, the Kruskal–Wallis test with Dunn *post hoc* testing for non-normal variables, and the chi-square or Fisher exact test for categorical variables; both prespecified pairwise *post hoc* comparisons (HFpEF vs. hypertensive controls and HFpEF vs. healthy volunteers) are reported. Familywise error across the prespecified CMR parameters was controlled using the Holm method, and the Benjamini–Hochberg false-discovery rate was applied in sensitivity analyses. The magnitude of between-group differences was expressed as the standardized mean difference (Cohen's d) with 95% confidence intervals (Figure 2). Diagnostic discrimination was quantified by the AUC with 95% confidence intervals derived by the DeLong method; pairwise AUC comparisons used the DeLong test for correlated curves; and exploratory operating points with the associated sensitivity, specificity, positive and negative predictive values at the observed prevalence, overall accuracy, and Youden index were obtained by maximizing the Youden statistic. These operating points were data-derived from the receiver operating characteristic curves of the present development cohort by maximization of the Youden index (J = sensitivity + specificity − 1) and were not adopted from external reference values, nor were they extracted from the coefficients of the multiparametric model, which used continuous standardized predictors rather than dichotomized inputs; because data-derived cutpoints are known to display optimistic performance in the sample from which they were obtained, they are reported for descriptive and hypothesis-generating purposes only. Because hypertension, diabetes, and atrial fibrillation were absent among healthy volunteers and produced complete or quasi-complete separation, the multivariable diagnostic model was fitted using Firth-penalized logistic regression and included the three prespecified cine markers (LAVI, LVMI, and GLS) with adjustment for age, sex, and body mass index; covariates exhibiting separation were retained only in sensitivity models, and continuous predictors were standardized so that odds ratios are expressed per standard deviation. Model performance was internally validated using 1,000 bootstrap resamples to derive an optimism-corrected AUC and a calibration slope, and both apparent and corrected estimates are reported. The 95% confidence interval for the optimism-corrected AUC was obtained from the 2.5th and 97.5th percentiles of the bootstrap distribution. A prespecified sensitivity analysis repeated the discrimination analyses using hypertensive controls as the sole comparator. Paired baseline-to-follow-up changes were assessed with the paired *t*-test or the Wilcoxon signed-rank test, expressed with 95% confidence intervals and the standardized paired effect size (Cohen's dz), and benchmarked against the MDC_95_; completers were compared with non-completers to evaluate attrition bias. For the *post hoc* stratification of the HFpEF cohort according to the cine CMR phenotype, defined as fulfillment of at least two of the three Youden-derived marker thresholds, between-subgroup comparisons used the Welch *t*-test for approximately normally distributed continuous variables, the Mann–Whitney U-test for skewed variables, and the Fisher exact test for categorical variables, and six-month changes in LAVI, GLS, and LVEF were compared between subgroups using the Welch *t*-test. Because this stratification was *post hoc* and exploratory, its *P-*values were not adjusted for multiplicity and are interpreted descriptively. There were no missing data for the cine parameters used to define the primary analysis. Two-sided *P*-values below 0.05 were considered statistically significant, and analyses were performed in R version 4.3.1 (R Foundation for Statistical Computing) using the pROC, rms, and logistf packages.

**Figure 2:**
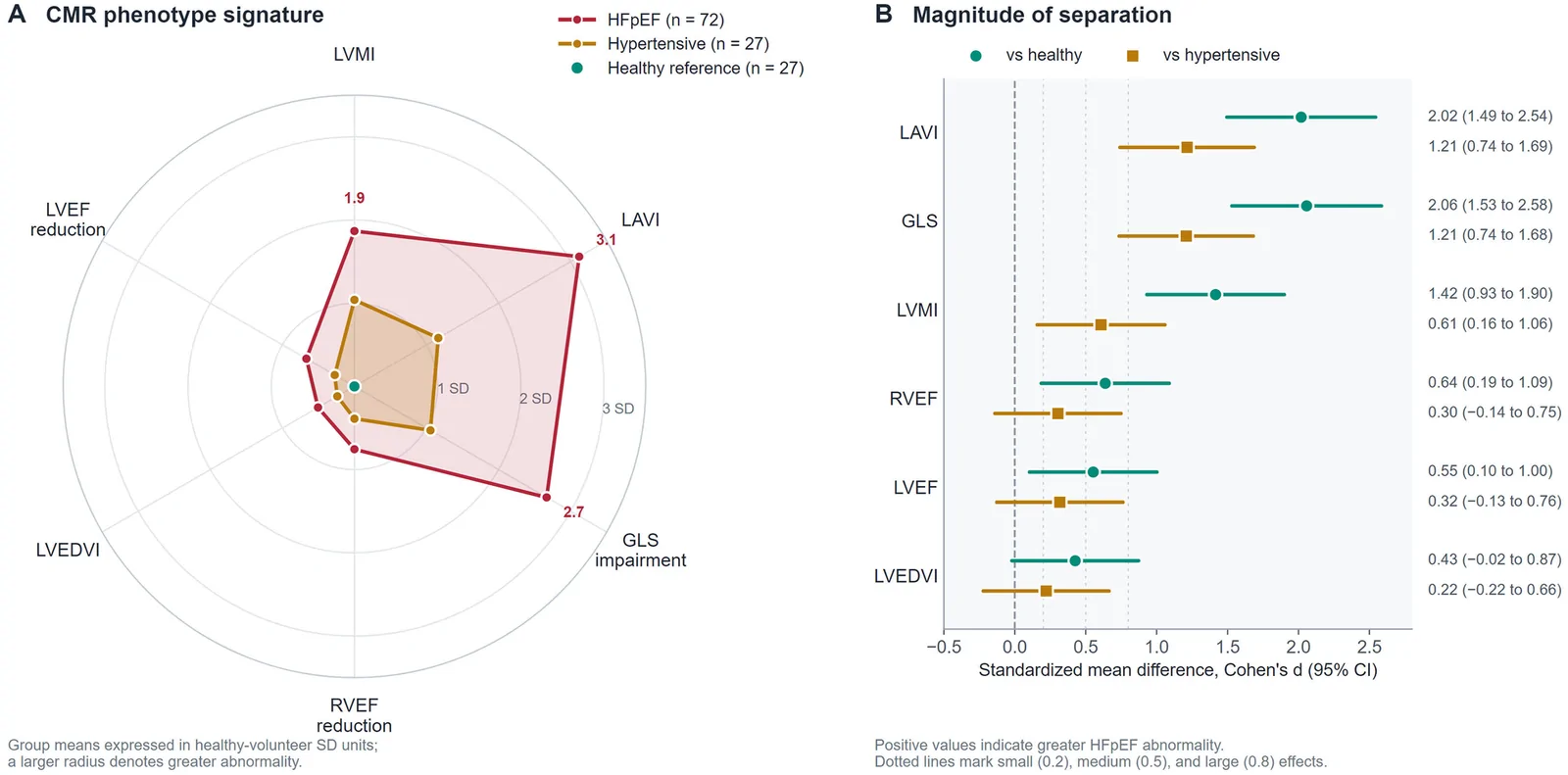
Cine CMR phenotype of HFpEF. **(A)** Radar plot of group mean values expressed in healthy-volunteer standard deviation units and oriented so that a larger radius denotes greater abnormality; the HFpEF profile expands along the LAVI, GLS impairment, and LVMI axes, the hypertensive profile is intermediate, and the healthy profile lies near the center. **(B)** Forest plot of standardized mean differences (Cohen's d) with 95% confidence intervals for each parameter relative to healthy volunteers and to hypertensive controls. The figure conveys the multivariate signature and the magnitude of separation, complementing the absolute values in Table 2.

## Results

### Participants and clinical profile

Of the adults identified during the study period, 126 met eligibility criteria and had analyzable baseline cine CMR examinations: 72 with adjudicated HFpEF, 27 hypertensive controls, and 27 healthy volunteers (Figure 1). Baseline clinical characteristics are detailed in Table 1. The three groups were well matched for age (HFpEF 68.1 ± 8.7 years, hypertensive 65.7 ± 9.1 years, healthy 64.9 ± 9.8 years; *P* = 0.241) and for sex distribution (women 61.1%, 51.9%, and 59.3%, respectively; *P* = 0.698), minimizing demographic confounding of the imaging comparison. Cardiometabolic burden, by contrast, was concentrated in the HFpEF cohort, which had the highest body mass index (28.4 ± 3.7 kg/m^2^; *P* < 0.001) and the highest prevalence of diabetes mellitus (36.1%), atrial fibrillation (26.4%), and chronic kidney disease, together with the lowest estimated glomerular filtration rate (Table 1). Hypertension was present in all hypertensive controls by definition and in 83.3% of patients with HFpEF but in no healthy volunteers. NT-proBNP was approximately fivefold higher in HFpEF than in hypertensive controls and an order of magnitude higher than in healthy volunteers [768 (402–1,314) vs. 146 (84–241) vs. 62 (39–98) pg/mL; *P* < 0.001], and patients with HFpEF more frequently received loop diuretics, renin–angiotensin system inhibitors, mineralocorticoid receptor antagonists, and sodium-glucose cotransporter 2 inhibitors. Despite these differences, echocardiographic LVEF was within the preserved range in every group and did not differ significantly (58.6% ± 4.1%, 60.2% ± 3.5%, and 61.4% ± 3.2%; *P* = 0.095), confirming that conventional systolic function did not separate HFpEF from its comparators and motivating the cine CMR analysis.

**Table 1:** Baseline clinical characteristics of the study population.

Variable	Overall (*N* = 126)	HFpEF (*n* = 72)	Hypertension (*n* = 27)	Healthy (*n* = 27)	*P*-value
Age, years	66.9 ± 9.3	68.1 ± 8.7	65.7 ± 9.1	64.9 ± 9.8	0.241
Female sex, *n* (%)	74 (58.7)	44 (61.1)	14 (51.9)	16 (59.3)	0.698
Body mass index, kg/m^2^	27.2 ± 3.8	28.4 ± 3.7	26.9 ± 3.4	24.1 ± 2.8	<0.001
Systolic blood pressure, mmHg	132 ± 15	138 ± 15	134 ± 12	121 ± 9	<0.001
Diastolic blood pressure, mmHg	78 ± 9	78 ± 9	80 ± 8	74 ± 7	0.012
Resting heart rate, bpm	70 ± 11	72 ± 12	70 ± 9	66 ± 8	0.041
Hypertension, *n* (%)	87 (69.0)	60 (83.3)	27 (100.0)	0 (0.0)	<0.001
Diabetes mellitus, *n* (%)	33 (26.2)	26 (36.1)	7 (25.9)	0 (0.0)	<0.001
Atrial fibrillation, *n* (%)	22 (17.5)	19 (26.4)	3 (11.1)	0 (0.0)	0.003
Estimated GFR, mL/min/1.73 m^2^	75 ± 18	68 ± 18	78 ± 14	88 ± 11	<0.001
NYHA class II/III, *n* (%)	-	47 (65)/25 (35)	-	-	-
NT-proBNP, pg/mL	248 (98–742)	768 (402–1,314)	146 (84–241)	62 (39–98)	<0.001
LVEF by echocardiography, %	59.5 ± 4.0	58.6 ± 4.1	60.2 ± 3.5	61.4 ± 3.2	0.095
Loop diuretic, *n* (%)	44 (34.9)	42 (58.3)	2 (7.4)	0 (0.0)	<0.001
RAAS inhibitor, *n* (%)	77 (61.1)	55 (76.4)	22 (81.5)	0 (0.0)	<0.001
MRA, *n* (%)	27 (21.4)	24 (33.3)	3 (11.1)	0 (0.0)	<0.001
SGLT2 inhibitor, *n* (%)	38 (30.2)	34 (47.2)	4 (14.8)	0 (0.0)	<0.001
Beta-blocker, *n* (%)	51 (40.5)	40 (55.6)	11 (40.7)	0 (0.0)	<0.001

GFR, glomerular filtration rate; LVEF, left ventricular ejection fraction; MRA, mineralocorticoid receptor antagonist; NT-proBNP, N-terminal pro-B-type natriuretic peptide; NYHA, New York Heart Association; RAAS, renin–angiotensin–aldosterone system; SGLT2, sodium-glucose cotransporter 2.

Values are mean ± SD, median (interquartile range), or *n* (%). NYHA class applies to the HFpEF cohort only.

**Table 2:** Baseline cine CMR parameters across the three study groups with pairwise comparisons.

Parameter	HFpEF (n = 72)	Hypertension (*n* = 27)	Healthy (*n* = 27)	*P* (HFpEF vs. HTN)	*P* (HFpEF vs. HC)
LVEDVI, mL/m^2^	72.4 ± 12.1	69.8 ± 10.8	67.5 ± 9.7	0.62	0.11
LVESVI, mL/m^2^	29.6 ± 6.4	27.7 ± 5.9	26.1 ± 4.8	0.45	0.08
LV stroke volume index, mL/m^2^	42.8 ± 8.1	42.1 ± 7.6	41.4 ± 6.9	0.79	0.55
LVEF by CMR, %	59.1 ± 4.2	60.4 ± 3.8	61.3 ± 3.3	0.34	0.09
LVMI, g/m^2^	76.8 ± 13.9	68.7 ± 11.6	58.5 ± 9.8	0.004	<0.001
LV mass-to-volume ratio, g/mL	1.07 ± 0.18	0.99 ± 0.15	0.87 ± 0.12	0.03	<0.001
LAVI, mL/m^2^	52.7 ± 11.4	39.6 ± 8.9	31.8 ± 6.7	<0.001	<0.001
RVEF, %	51.4 ± 6.1	53.2 ± 5.4	55.1 ± 4.9	0.18	0.04
GLS, %	−14.2 ± 2.5	−17.1 ± 2.1	−19.0 ± 1.8	<0.001	<0.001

GLS, global longitudinal strain; HC, healthy controls; HTN, hypertension; LAVI, left atrial volume index; LVEDVI and LVESVI, left ventricular end-diastolic and end-systolic volume index; LVEF, left ventricular ejection fraction; LVMI, left ventricular mass index; RVEF, right ventricular ejection fraction.

Values are mean ± SD. Pairwise *P*-values are *post hoc* (Tukey or Dunn) with Holm adjustment. GLS is reported as a negative percentage; less negative values indicate worse longitudinal deformation. Interobserver reproducibility: intraclass correlation 0.95 (LAVI) and 0.91 (GLS); within-subject coefficient of variation 4.8% and 6.2%.

### Reproducibility of cine CMR markers

Interobserver reproducibility of the markers underpinning the diagnostic and serial analyses was high. Intraclass correlation coefficients were 0.95 for LAVI and 0.91 for GLS, within-subject coefficients of variation were 4.8% and 6.2%, and Bland–Altman analysis demonstrated negligible systematic bias (0.4 mL/m^2^ for LAVI and 0.1 percentage points for GLS) with narrow limits of agreement. The resulting MDC_95_ values of 2.8 mL/m^2^ for LAVI and 1.2 percentage points for GLS provided objective thresholds against which the 6-month changes were judged, thereby supporting the use of these markers for serial monitoring.

### Baseline cine CMR phenotype

The cine CMR phenotype of HFpEF was defined by remodeling and impaired mechanics rather than by chamber dilation or reduced ejection fraction (Table 2, Figure 2). Left ventricular end-diastolic and end-systolic volume indices, indexed stroke volume, and CMR-derived LVEF did not differ significantly among the groups, and right ventricular ejection fraction was       modestly lower in HFpEF than in healthy volunteers (51.4 ± 6.1% vs. 55.1 ± 4.9%; *P* = 0.04). In contrast, LVMI was significantly higher in HFpEF than in both hypertensive controls and healthy volunteers (76.8 ± 13.9 vs. 68.7 ± 11.6 and 58.5 ± 9.8 g/m^2^; pairwise *P* = 0.004 and *P* < 0.001), the left ventricular mass-to-volume ratio was correspondingly increased, LAVI showed the greatest structural separation of any parameter (52.7 ± 11.4 vs. 39.6 ± 8.9 and 31.8 ± 6.7 mL/m^2^; pairwise *P* < 0.001 for both), and GLS was markedly less negative despite preserved LVEF (−14.2 ± 2.5% vs. −17.1 ± 2.1% and −19.0 ± 1.8%; pairwise *P* < 0.001 for both). The multivariate signature is illustrated in Figure 2A, where the HFpEF profile extends along the LAVI, GLS, and LVMI axes while remaining close to the healthy reference for volumes and ejection fraction, and the hypertensive profile occupies an intermediate position. The corresponding standardized effect sizes (Figure 2B) were very large relative to healthy volunteers for LAVI (d 2.02, 95% confidence interval 1.49–2.54) and GLS (d 2.06, 95% confidence interval 1.53–2.58) and large for LVMI (d 1.42), and they remained large for LAVI and GLS (d 1.21 for both) and moderate for LVMI (d 0.61) even against the more demanding hypertensive comparator, whereas effect sizes for LVEF and ventricular volumes were small (d less than 0.6). Preserved ejection fraction therefore did not reflect preserved myocardial mechanics.

### Diagnostic discrimination and multiparametric model

The discriminatory performance of each cine marker is summarized in Table 3 and Figure 3. Among single parameters, GLS and LAVI were the strongest discriminators of HFpEF (apparent AUC 0.85, 95% confidence interval 0.77–0.91, and 0.83, 95% confidence interval 0.75–0.90, respectively), substantially exceeding LVMI (0.74, 95% confidence interval 0.65–0.83). At its Youden-optimal operating point, GLS greater than −15.8% provided 80.6% sensitivity, 79.2% specificity, 83.8% positive predictive value, 75.4% negative predictive value, and 80.0% overall accuracy, while LAVI greater than 44 mL/m^2^ provided 79.2% sensitivity and 76.4% specificity with comparable predictive values (Table 3). The multiparametric model combining LAVI, LVMI, and GLS achieved the highest apparent discrimination (AUC 0.87, 95% confidence interval 0.80–0.93; sensitivity 83.3%; specificity 81.1%; accuracy 82.4%; Youden index 0.64). After 1,000-sample bootstrap internal validation, the optimism-corrected AUC was 0.84 with a calibration slope of 0.88, indicating only modest overfitting (Figure 3B). On formal comparison, the model did not differ significantly from GLS alone (difference in AUC 0.02; DeLong *P* = 0.34) but significantly exceeded LVMI (*P* = 0.01). In the Firth-penalized regression, larger LAVI (adjusted odds ratio per standard deviation 2.4, 95% confidence interval 1.5–3.9) and less negative GLS (odds ratio 2.1, 95% confidence interval 1.3–3.5) remained independent predictors of HFpEF after adjustment for age, sex, and body mass index, whereas LVMI was directional but weaker (odds ratio 1.4, 95% confidence interval 0.9–2.2). In the prespecified sensitivity analysis restricted to hypertensive controls, discrimination was consistently lower (AUC 0.77 for LAVI, 0.79 for GLS, and 0.81 for the model), confirming that inclusion of healthy volunteers in the combined comparator inflated apparent performance. These results represent apparent and internally corrected discrimination within a single-center development cohort and require external validation before clinical application.

**Table 3:** Diagnostic performance of cine CMR markers and the multiparametric model for HFpEF.

Marker	AUC (95% CI)	Cut point	Sens, %	Spec, %	PPV, %	NPV, %	Acc, %
LVMI	0.74 (0.65–0.83)	>71 g/m^2^	68.1	71.7	76.2	62.8	69.6
LAVI	0.83 (0.75–0.90)	>44 mL/m^2^	79.2	76.4	81.7	73.4	78.0
GLS	0.85 (0.77–0.91)	>−15.8%	80.6	79.2	83.8	75.4	80.0
Model (apparent)	0.87 (0.80–0.93)	—	83.3	81.1	85.4	78.5	82.4
Model (optimism-corrected)	0.84 (0.76–0.91)	—	—	—	—	—	—

Acc, accuracy; AUC, area under the receiver operating characteristic curve; CI, confidence interval; NPV, negative predictive value; PPV, positive predictive value; Sens, sensitivity; Spec, specificity.

Operating points maximize the Youden index (J = 0.40 for LVMI, 0.56 for LAVI, 0.60 for GLS, and 0.64 for the model). Predictive values are reported at the observed prevalence of 57.1%. The model combined LAVI, LVMI, and GLS using Firth-penalized logistic regression; the optimism-corrected AUC and calibration slope (0.88) were derived from 1,000 bootstrap resamples. DeLong comparisons: model vs. GLS *P* = 0.34; model vs. LVMI *P* = 0.01. In the sensitivity analysis restricted to hypertensive controls, AUCs were 0.77 (LAVI), 0.79 (GLS), and 0.81 (model). Values are apparent single-center estimates and require external validation.

**Figure 3:**
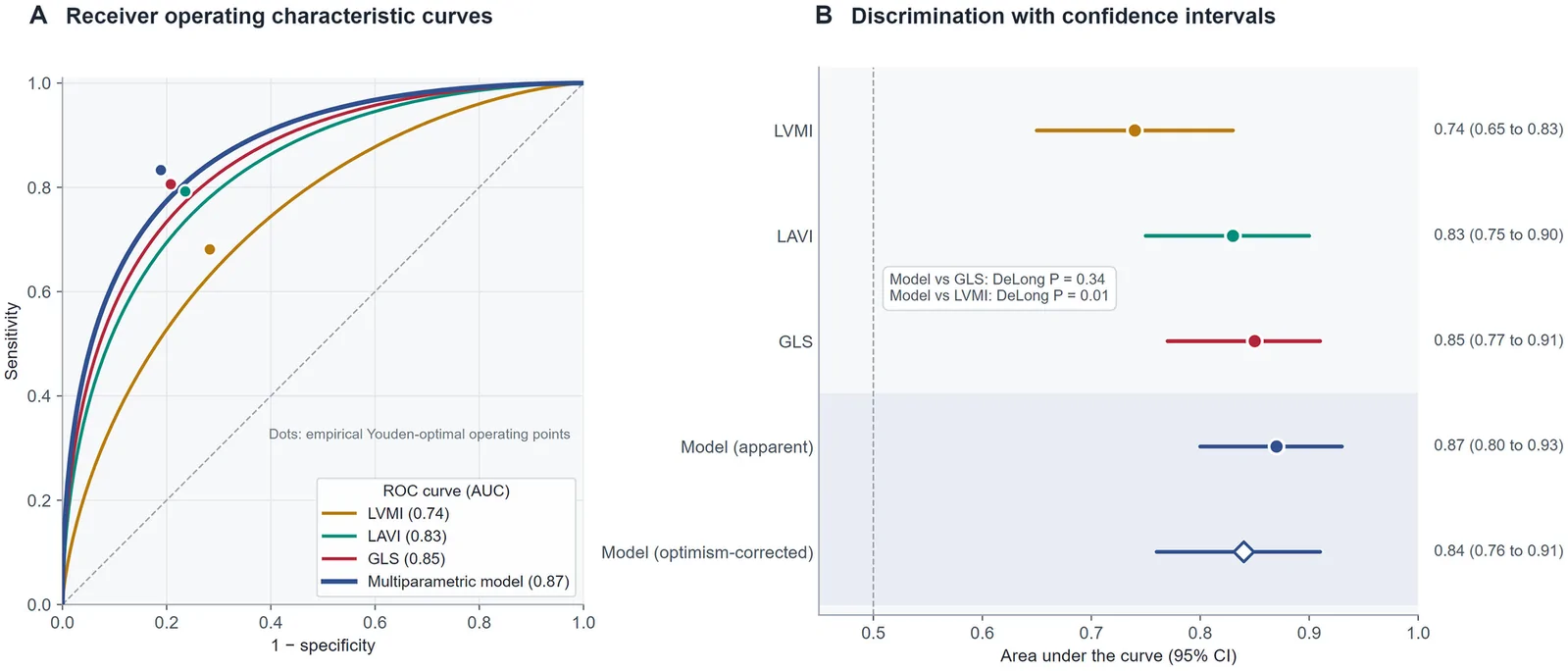
Diagnostic discrimination for HFpEF. **(A)** Receiver operating characteristic curves for LAVI, LVMI, GLS, and the multiparametric model. For visual presentation, ROC curves in **(A)** were smoothed using a binormal fit; the marked Youden operating points were derived from the empirical ROC curves reported in Table 3. **(B)** Areas under the curve with 95% confidence intervals for each marker and for the model before and after bootstrap optimism correction; DeLong comparisons of the model with GLS and with LVMI are annotated. Estimates represent apparent and internally corrected single-center performance and require external validation.

### Six-month serial assessment

Forty-six of the 72 patients with HFpEF (64%) had repeat cine CMR available at a median of 6 months as part of clinical follow-up (Figure 1), and the remaining 26 had no repeat imaging available for the following reasons: withdrawal of consent for further imaging in eight patients, an interval contraindication in five (permanent cardiac device implantation in three, marked claustrophobia in one, and physician-led deferral of advanced imaging after a renal deterioration in one), scanner availability constraints or out-of-region referrals precluding rescheduling within the intended 6-month reassessment window in seven, and loss to imaging follow-up despite continuing clinical care in six. Completers and non-completers did not differ significantly in age, sex, body mass index, NT-proBNP, or baseline LAVI or GLS (all *P* > 0.20), suggesting that substantial attrition bias was unlikely, although selection related to unmeasured clinical factors cannot be excluded. The imaging response over follow-up was selective and is detailed in Table 4 and Figure 4. LAVI decreased from 51.3 ± 10.1 to 46.5 ± 10.4 mL/m^2^, a mean paired reduction of 4.8 mL/m^2^ (95% confidence interval −5.6 to −4.1; *P* < 0.001) corresponding to a relative reduction of 9.4% and a standardized paired effect size (dz) of 1.85; this change was 1.7 times the LAVI MDC_95_ (Figure 4C). GLS became more negative, improving from −14.1 ± 2.3% to −15.4 ± 2.6%, a mean paired change of −1.3 percentage points (95% confidence interval −1.6 to −1.0; *P* < 0.001; dz 1.44) that exceeded the GLS MDC_95_ by a factor of 1.1. Both changes therefore exceeded the prespecified thresholds for measurement error (Figures 4A,4B), whereas CMR-derived LVEF remained within the preserved range without significant change, indicating that the observed improvements reflected atrial unloading and enhanced longitudinal mechanics rather than a change in global systolic function. Because treatment was individualized rather than randomized and the substudy included no untreated comparator, these changes cannot be attributed to any single intervention and may be influenced in part by regression to the mean.

**Table 4:** Six-month paired cine CMR changes among HFpEF patients who completed repeat imaging (*n* = 46).

Parameter	Baseline	6 months	Mean change (95% CI)	Change, %	d_z	MDC_95_	>MDC_95_
LAVI, mL/m^2^	51.3 ± 10.1	46.5 ± 10.4	−4.8 (−5.6 to −4.1)	−9.4	1.85	2.8	Yes
GLS, %	−14.1 ± 2.3	−15.4 ± 2.6	−1.3 (−1.6 to −1.0)	+9.2	1.44	1.2	Yes

d_z, standardized paired effect size (Cohen's dz); MDC_95_, minimal detectable change at the 95% level (2.8 mL/m^2^ for LAVI; 1.2 percentage points for GLS).

Values are mean ± SD unless otherwise stated. A negative LAVI change indicates lower indexed left atrial volume, and a more negative GLS indicates improved longitudinal deformation (reported here as a positive percentage improvement). Paired comparisons used the paired *t*-test. CMR-derived LVEF remained within the preserved range without significant change. Treatment was individualized rather than randomized.

**Figure 4:**
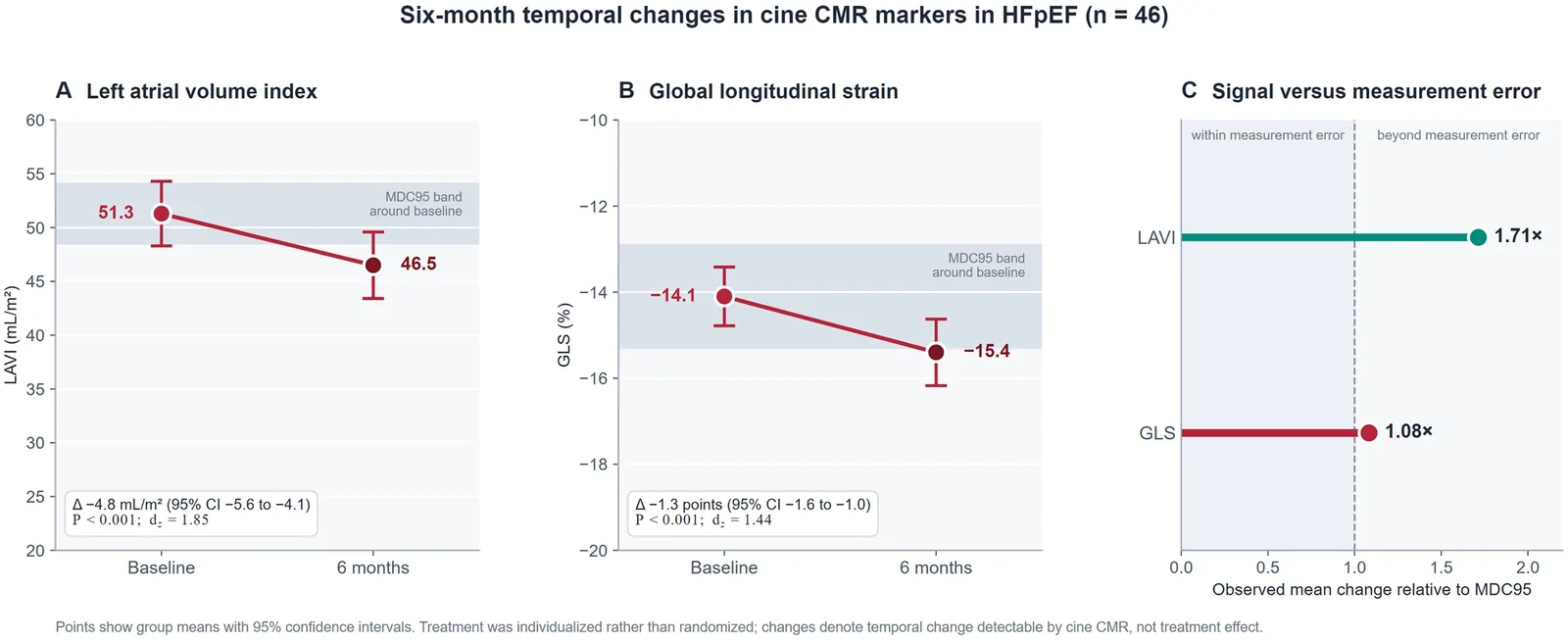
Six-month temporal changes in cine CMR markers in HFpEF   (*n* = 46). **(A,B)** Group means with 95% confidence intervals at baseline and follow-up for LAVI and GLS; the shaded band shows the minimal detectable change (MDC_95_) around the baseline mean, and the annotation reports the mean paired change, its confidence interval, the *P-*value, and the standardized paired effect size. **(C)** Observed mean change expressed relative to the MDC_95_; values exceeding 1.0 indicate change beyond measurement error. Treatment was individualized rather than randomized.

### CMR phenotype stratification within adjudicated HFpEF

Because the conventional clinical HFpEF definition and the cine CMR signature may capture overlapping but non-identical aspects of the syndrome, we performed a *post hoc* stratification of the 72 adjudicated HFpEF patients according to the presence of the cine CMR phenotype, defined as fulfillment of at least two of the three prespecified cine markers using the *post hoc* Youden-derived operating points (LAVI greater than 44 mL/m^2^, GLS less negative than −15.8%, and LVMI greater than 71 g/m^2^) reported in Table 3. Fifty-six patients (77.8%) exhibited the CMR phenotype, and 16 (22.2%) did not, indicating substantial but incomplete concordance between the clinical and imaging classifications. Compared with phenotype-absent HFpEF, phenotype-present patients showed a markedly higher biomarker burden [median NT-proBNP 892 [interquartile range 492–1,502 vs. 462 (218–843) pg/mL; *P* < 0.001] together with numerically higher frequencies of NYHA class III symptoms, atrial fibrillation, and mineralocorticoid receptor antagonist use, although none of these categorical comparisons reached conventional statistical significance at the current sample size (NYHA class III symptoms 39.3% vs. 18.8%, *P* = 0.15; atrial fibrillation 32.1% vs. 6.3%, *P* = 0.053; mineralocorticoid receptor antagonist use 39.3% vs. 12.5%, *P* = 0.070), and a modestly lower estimated glomerular filtration rate (Supplementary Table S1). Among the 46 patients who completed paired follow-up imaging, patients with the CMR phenotype at baseline experienced a significantly larger absolute reduction in LAVI (mean paired change −5.6 vs. −2.7 mL/m^2^, *P* = 0.023) and a directionally greater improvement in GLS (−1.5 vs. −0.8 percentage points, *P* = 0.084) over 6 months, whereas the differential response to individually optimized therapy is reported as exploratory only because treatment was not randomized. These stratified comparisons should be interpreted as descriptive observations from a single-center cohort, but they suggest that the CMR signature adds an analytic layer that conventional clinical criteria do not provide on their own.

## Discussion

This retrospective single-center analysis supports a pragmatic, contrast-free cine CMR approach to HFpEF phenotyping. The central observation is that HFpEF was distinguished not by reduced ejection fraction or chamber dilation but by a reproducible pattern of left atrial enlargement, increased left ventricular mass, and impaired longitudinal deformation. LAVI and GLS provided the strongest discrimination, with large standardized effect sizes, and a multiparametric model combining LAVI, LVMI, and GLS yielded the highest apparent accuracy. These findings reflect the contemporary understanding that HFpEF is governed less by global systolic failure than by abnormal filling pressures, atrial myopathy, myocardial stiffness, and subtle systolic dysfunction that ejection fraction does not capture (2, 6, 7). It is nonetheless essential to recognize that HFpEF is a highly heterogeneous syndrome whose pathophysiology extends beyond structural remodeling and myocardial mechanics to encompass metabolic, neurohormonal, autonomic, renal, and inflammatory abnormalities (37), and that cine CMR captures a clinically important but partial component of this multidimensional phenotype rather than a comprehensive appraisal of the underlying disease biology.

The prominence of LAVI is biologically coherent. Left atrial enlargement integrates cumulative exposure to elevated left-sided filling pressures rather than a single hemodynamic moment, and CMR assessment of the atrium is less dependent on acoustic windows than echocardiography (14). Our results align with reports that left atrial size and strain identify HFpEF as accurately as, or better than, several ventricular indices (15, 38), including among hypertensive patients in whom the distinction is most demanding (16), and with the recognition that atrial roof remodeling is a distinctive structural feature (17). The community-based observation that longitudinal decline in left atrial function precedes incident HFpEF further supports atrial remodeling as a central, and potentially early, imaging signature rather than an incidental finding (21).

The GLS findings add complementary information. Preserved LVEF can coexist with impaired longitudinal deformation because ejection fraction is strongly influenced by chamber geometry, radial thickening, and loading conditions, whereas longitudinal subendocardial fibers are vulnerable to microvascular dysfunction, fibrosis, and increased wall stress (6). Feature-tracking GLS is impaired in HFpEF, correlates with invasive relaxation indices, and carries prognostic weight despite normal ejection fraction (18–20, 39), and the combined assessment of atrial and ventricular strain refines risk stratification (40). The lower right ventricular ejection fraction observed in HFpEF, reaching nominal statistical significance against healthy volunteers despite a modest effect size, is consistent with the recognized contribution of right ventricular involvement to the syndrome (41). Interpretation of strain nonetheless requires caution, because feature-tracking values vary by vendor, software, contouring, and temporal resolution; we therefore standardized acquisition, blinded analysis, and quantified reproducibility, observing high intraclass correlation and low within-subject variability that support the serial application of these markers.

The multiparametric model outperformed the weakest single marker but did not significantly exceed GLS alone, and its apparent discrimination declined after optimism correction. This pattern argues for restraint: the model is best regarded as hypothesis-generating, integrating complementary domains of pressure-overload remodeling, chronic filling-pressure burden, and impaired mechanics, rather than as a deployable diagnostic rule. Critically, discrimination was lower when the comparator was restricted to hypertensive controls, the population that most closely resembles real diagnostic uncertainty, indicating that performance against a mixed comparator that includes healthy volunteers is optimistic. External validation in cohorts enriched for unexplained dyspnea, obesity, atrial fibrillation, and hypertension, with external calibration assessment, is essential before any clinical use. Because clinicians rarely need imaging support to distinguish HFpEF from unequivocally healthy individuals, the clinically meaningful discriminatory task is between HFpEF and hypertensive cardiac remodeling; in the present sensitivity analysis, the multiparametric model reached an AUC of 0.81 against hypertensive controls (0.79 for GLS and 0.77 for LAVI), which reflects the substantial overlap in pressure-overload hypertrophy and atrial enlargement shared between the two conditions and reinforces the view that external validation should preferentially enrich comparator cohorts with treated hypertension, obesity, atrial fibrillation, and unexplained exertional dyspnea rather than unselected healthy volunteers.

The serial findings are clinically relevant but must be interpreted conservatively. Contemporary HFpEF management combines decongestion, blood pressure control, rhythm management, sodium-glucose cotransporter 2 inhibition, and comorbidity-directed care (28–35), and clinicians increasingly require objective markers of response beyond symptoms and natriuretic peptide levels. The observed reduction in LAVI and improvement in GLS, both exceeding the minimal detectable change, suggest that cine CMR can capture atrial unloading and improved mechanics even when ejection fraction is static, consistent with imaging evidence of reverse remodeling during sodium-glucose cotransporter 2 inhibition (36) and with the responsiveness of atrial function demonstrated in dedicated CMR HFpEF studies (42). Given these design constraints, we would emphasize that the 6-month findings are best interpreted as evidence that cine CMR can detect temporal changes in atrial size and longitudinal deformation over a clinically meaningful interval, rather than as evidence of treatment-induced reverse remodeling attributable to any specific pharmacological intervention. Spontaneous variation, regression toward the mean, and time-related improvements in comorbidities such as blood pressure, volume status, and heart rate cannot be separated from any pharmacological effect within an individualized management framework. Because therapy was individualized rather than randomized and the substudy lacked an untreated comparator, these changes cannot be attributed to any single intervention and may be influenced in part by regression to the mean; comparable completer and non-completer characteristics argue against major attrition bias but do not eliminate it.

Several limitations warrant consideration. The study was conducted at a single center with a moderate sample size, and follow-up imaging was available for 46 of 72 patients. Advanced tissue characterization was deliberately omitted, so myocardial fibrosis, extracellular volume expansion, epicardial adipose burden, and ischemic reserve, all implicated in HFpEF pathophysiology, were not evaluated (22, 43, 44); cine markers should therefore be viewed as complementary to, and not a substitute for, comprehensive CMR that includes late gadolinium enhancement, native T1 mapping, extracellular volume quantification, and, where indicated, stress perfusion imaging, as well as established echocardiographic diagnostic frameworks (23, 24). Whenever the clinical question requires tissue-level information such as the detection of interstitial fibrosis, infiltration, ischemia, or an alternative cardiomyopathic etiology, a comprehensive multiparametric CMR examination remains the appropriate investigation. Finally, although readers were blinded, the most discriminating features are visually conspicuous, so blinding cannot be considered absolute. Despite these constraints, the analysis addresses a practical question with broad applicability: whether standard cine CMR, available in most laboratories, can reveal the structural and functional HFpEF phenotype that ejection fraction alone misses. The answer from this cohort is affirmative but not definitive.

The clinical positioning of these findings deserves careful articulation. In the first instance, we envision cine-derived LAVI and GLS as supportive phenotyping markers in patients who are already undergoing CMR for other indications, so that a routinely acquired cine dataset can be leveraged to characterize the atrioventricular remodeling and longitudinal mechanics that ejection fraction alone does not reveal, without adding scan time, contrast agent, or additional postprocessing complexity. Beyond this primary role, we propose cine CMR as an adjunct examination following an inconclusive echocardiographic assessment, particularly in patients with suboptimal acoustic windows, marked obesity, chronic pulmonary disease, or discordant HFA-PEFF scoring in whom the clinical suspicion of HFpEF remains high. In neither scenario do we propose that cine CMR should supplant the current echocardiography-first pathway or the comprehensive CMR examination when tissue characterization is clinically indicated; rather, we anticipate a stepwise use of imaging in which the routine cine sequence contributes an additional layer of phenotypic information at low incremental cost.

Beyond diagnostic discrimination, the cine CMR phenotype appears to identify a subset of adjudicated HFpEF patients whose imaging profile is coherent with more advanced atrial myopathy, greater hypertrophic remodeling, and more pronounced longitudinal mechanical impairment, and whose clinical profile is associated with a higher biomarker burden and a numerically higher symptomatic burden. That patients with the CMR phenotype at baseline also exhibited a significantly larger reduction in LAVI and a directionally greater improvement in GLS over 6 months is consistent with a more advanced phenotype showing greater temporal change in selected CMR markers. These observations are compatible with the hypothesis that cine CMR does more than confirm the clinical diagnosis, enriching phenotyping by distinguishing a structurally advanced subgroup from patients who meet clinical criteria yet display a less prominent imaging signature. Whether this cine-defined stratification carries independent prognostic weight or predicts differential response to guideline-directed medical therapy will require confirmation in multicenter cohorts with prospective outcome adjudication.

## Conclusions

Routine cine CMR identified a coherent HFpEF phenotype characterized by left atrial enlargement, increased left ventricular mass, and impaired longitudinal deformation despite preserved ejection fraction. LAVI and GLS were the strongest cine-derived markers, and their integration with LVMI improved apparent discrimination, although not beyond GLS alone after optimism correction. Serial imaging showed that LAVI and GLS, unlike LVEF, changed meaningfully over 6 months, an observation best interpreted as evidence of temporal detectability by cine CMR rather than as proof of treatment-induced reverse remodeling. Cine CMR is a practical adjunct to established HFpEF diagnostic pathways and warrants multicenter validation with standardized acquisition, reproducibility testing, and externally evaluated, calibrated prediction models.

## Data Availability

The original contributions presented in the study are included in the article/Supplementary Material, further inquiries can be directed to the corresponding author.
