# Supplementary material for: Routine cine cardiovascular magnetic resonance for clinical phenotyping and serial assessment of heart failure with preserved ejection fraction: a retrospective single-center clinical analysis
Source: Front Cardiovasc Med. 2026 Oct 19;13:1965415. doi: 10.3389/fcvm.2026.1965415 (PMC13724410; doi:10.3389/fcvm.2026.1965415)
Supplement: Supplementary file 1 [file Table1.docx]

**Supplementary Table S1.** Clinical and cine cardiovascular magnetic resonance characteristics of the adjudicated HFpEF cohort stratified by the presence or absence of the cine CMR phenotype (n = 72).

| **Variable** | **CMR phenotype present (n = 56)** | **CMR phenotype absent (n = 16)** | **P value** |
| --- | --- | --- | --- |
| Age, years | 68.6 ± 8.4 | 66.4 ± 9.8 | 0.42 |
| Female sex, n (%) | 34 (60.7) | 10 (62.5) | 1.00 |
| Body mass index, kg/m² | 28.7 ± 3.5 | 27.3 ± 4.3 | 0.25 |
| Systolic blood pressure, mmHg | 139 ± 15 | 134 ± 14 | 0.23 |
| Diabetes mellitus, n (%) | 22 (39.3) | 4 (25.0) | 0.38 |
| Atrial fibrillation, n (%) | 18 (32.1) | 1 (6.3) | 0.053 |
| Estimated GFR, mL/min/1.73 m² | 66 ± 17 | 74 ± 19 | 0.14 |
| NYHA class II, n (%) | 34 (60.7) | 13 (81.3) | 0.15 |
| NYHA class III, n (%) | 22 (39.3) | 3 (18.8) | 0.15 |
| NT-proBNP, pg/mL, median (IQR) | 892 (492–1502) | 462 (218–843) | <0.001 |
| Loop diuretic, n (%) | 36 (64.3) | 6 (37.5) | 0.084 |
| SGLT2 inhibitor, n (%) | 29 (51.8) | 5 (31.3) | 0.17 |
| MRA, n (%) | 22 (39.3) | 2 (12.5) | 0.070 |
| Baseline LAVI, mL/m² | 55.0 ± 10.4 | 44.9 ± 9.6 | 0.001 |
| Baseline LVMI, g/m² | 80.1 ± 12.9 | 64.9 ± 10.8 | <0.001 |
| Baseline GLS, % | −13.4 ± 2.2 | −17.1 ± 1.8 | <0.001 |
| Baseline LVEF (CMR), % | 58.9 ± 4.3 | 59.9 ± 3.8 | 0.38 |
| Follow-up cine CMR available, n (%) | 37 (66.1) | 9 (56.3) | 0.56 |
| Change in LAVI at 6 months, mL/m² | −5.6 (−6.5 to −4.7) | −2.7 (−4.3 to −1.1) | 0.023 |
| Change in GLS at 6 months, pp | −1.5 (−1.8 to −1.2) | −0.8 (−1.4 to −0.2) | 0.084 |
| Change in LVEF at 6 months, % | 0.4 ± 2.9 | 0.6 ± 2.6 | 0.84 |

*Values are mean ± standard deviation, median (interquartile range), or n (%). The cine CMR phenotype was defined as fulfilment of at least two of the three post hoc Youden-derived thresholds (left atrial volume index greater than 44 mL/m², global longitudinal strain less negative than −15.8%, and left ventricular mass index greater than 71 g/m²) applied to the 72 adjudicated HFpEF patients. Between-group baseline comparisons used the Welch t test for continuous variables, the Mann-Whitney U test for skewed distributions, and the Fisher exact test for categorical variables; six-month change comparisons used the Welch t test. Six-month LAVI and GLS changes are presented as mean paired change (95% CI), and the six-month LVEF change as mean ± standard deviation. Because the phenotype-absent subgroup was small (n = 16), several categorical differences were numerically apparent but did not reach conventional statistical significance and should be interpreted descriptively. GFR, glomerular filtration rate; GLS, global longitudinal strain; IQR, interquartile range; LAVI, left atrial volume index; LVEF, left ventricular ejection fraction; LVMI, left ventricular mass index; MRA, mineralocorticoid receptor antagonist; NT-proBNP, N-terminal pro-B-type natriuretic peptide; NYHA, New York Heart Association; pp, percentage points; SGLT2, sodium-glucose cotransporter 2. Values represent apparent single-center estimates from the development cohort and are hypothesis-generating.*
